# Exosomes Derived from Mesenchymal Stem Cells

**DOI:** 10.3390/ijms15034142

**Published:** 2014-03-07

**Authors:** Bo Yu, Xiaomin Zhang, Xiaorong Li

**Affiliations:** Department of the Uveitis & Ocular Immunology, Tianjin Medical University Eye Hospital & Eye Institute, No. 251 Fukang Road, Nankai District, Tianjin 300384, China; E-Mail: yubo4950@126.com

**Keywords:** mesenchymal stem cell, exosome, protain, miRNA

## Abstract

The functional mechanisms of mesenchymal stem cells (MSCs) have become a research focus in recent years. Accumulating evidence supports the notion that MSCs act in a paracrine manner. Therefore, the biological factors in conditioned medium, including exosomes and soluble factors, derived from MSC cultures are being explored extensively. The results from most investigations show that MSC-conditioned medium or its components mediate some biological functions of MSCs. Several studies have reported that MSC-derived exosomes have functions similar to those of MSCs, such as repairing tissue damage, suppressing inflammatory responses, and modulating the immune system. However, the mechanisms are still not fully understood and the results remain controversial. Compared with cells, exosomes are more stable and reservable, have no risk of aneuploidy, a lower possibility of immune rejection following *in vivo* allogeneic administration, and may provide an alternative therapy for various diseases. In this review, we summarize the properties and biological functions of MSC-derived exosomes and discuss the related mechanisms.

## 1. Introduction

Cells are known to secrete a large variety of vesicles into the extracellular space, of which exosomes have received the most attention in recent years. They were originally thought to be necessary for the clearance of unneeded proteins from cells [1], and the current opinion is that exosomes are specifically secreted vesicles involved in intercellular communication. Exosomes were also found to transport RNA in later research. Moreover, exosomes are secreted by all cell types and are present in body fluids such as blood, urine, and breast milk.

Mesenchymal stem cells (MSCs) are self-renewing, multipotent progenitors that can be isolated from various tissues. They are widely tested in clinical trials because of their multiple biological functions including multilineage differentiation, tissue-repair promotion, anti-inflammatory medication, immunosuppression, and neuroprotection. In terms of the mechanisms underlying these biological functions, it was originally thought that MSCs home to injured tissues, differentiate, and replace damaged cells. However, subsequent research showed that MSC engraftment and differentiation at injury sites are very low and transient [2]. Currently, it is proposed that MSCs exert their therapeutic effects mainly through secreted trophic factors. Because exosomes are involved in cell-to-cell communication, some researchers hypothesize that they are the paracrine effectors of MSCs. They have been tested in various disease models and the results have revealed that their functions are similar to those of MSCs, such as reducing the size of myocardial infarctions, facilitating the repair of kidney injury, modulating immune responses, and promoting tumor growth. In this review, we summarize the current knowledge on the composition, functions, and isolation strategies of MSC-derived exosomes, and discuss their potential therapeutic applications.

## 2. Basic Characteristics and Biological Functions of MSCs

### 2.1. Basic Characteristics

MSCs are one of the most easily accessible primary cells and can be easily harvested from a large variety of tissues, such as adipose tissue, umbilical cord blood, liver, amniotic fluid, and placenta, as well as dental pulp, and other sources [3,4]. These cells can differentiate into both mesenchymal and non-mesenchymal cell lineages [5]. The ease of isolation and specialized biological functions of MSCs have made them a popular choice for cell therapy in preclinical and clinical trials.

### 2.2. Biological Functions

#### 2.2.1. Multilineage Differentiation Potential

MSCs are characterized by their potential for differentiation into multiple mesenchymal lineages, such as bone, fat, cartilage, and muscle, and non-mesenchymal cell lineages, such as neurons, glial cells, and hepatocytes. These properties have made MSCs a seed cell type in tissue engineering and regenerative medicine including reconstruction of bone and cartilage, nerve regeneration, and vascular tissue repair [6,7].

#### 2.2.2. Promotion of Tissue Repair

MSCs have the ability to migrate to injured tissues and release cytokines, inflammatory mediators, extracellular matrix components, and antimicrobial proteins to generate an appropriate microenvironment for tissue repair. MSCs have been reported to play important roles in lung and kidney injury as well as cartilage and long bone repair [8]. In myocardial infarction models, MSC transplantation has resulted in a reduction of the infarct size, improvement of the left ventricular ejection fraction, and increases of vascular density and myocardial perfusion [9]. In corneal and retinal injury models, the application of MSCs to the injured tissue has been demonstrated to improve wound healing [10].

#### 2.2.3. Immunosuppression

Over the past decade, MSCs have been shown to possess a broad spectrum of immunoregulatory capabilities. Accumulated data have demonstrated that the proliferation of T cells stimulated with either polyclonal mitogens or antigens is inhibited by MSCs [11]. In addition, MSCs inhibit the function of other immune cell subpopulations in adaptive and innate immunity, including professional antigen-presenting cells such as B cells, dendritic cells, and macrophages, as well as natural killer cells [12,13]. MSCs are poorly immunogenic and have the ability to influence the cytokine secretion profile of T-cell subsets. These properties serve as the rationale for clinical trials of MSCs in graft-versus-host disease [14], and support further research of MSC application in organ transplantation, including skin, kidney, heart, and corneal transplantation [15], and autoimmune diseases such as multiple sclerosis, uveitis, inflammatory bowel disease, arthritis, systemic lupus erythematosus, and diabetes mellitus [16–18].

#### 2.2.4. Neuroprotective Effect

MSCs can transdifferentiate into neural cells and secrete various neurotrophic and anti-inflammatory factors following transplantation, which confers strong neuroprotective effects in models of amyotrophic lateral sclerosis, multiple sclerosis, Parkinson’s disease, and glaucoma [19].

### 2.3. Mechanisms

Although various functions have been reported for MSCs, the underlying mechanisms are only partially understood. It has been hypothesized that transplanted MSCs can differentiate into other cell types to repair tissue damage. However, this hypothesis has proved to be unreliable by some researchers [20]. It is generally accepted that membrane-bound and soluble factors are more important. Recently, attention has been paid to the exosome, a membrane-bound vesicle isolated from MSC culture supernatants.

## 3. Basic Characteristics of Exosomes

### 3.1. History and Concept

The secretion of nanovesicles during maturation of sheep reticulocytes was discovered in the 1980s [21]. These vesicles were named exosomes and thought to be necessary to remove unneeded proteins from cells. Thereafter, many cell types were found to secrete exosomes, including B and T cells, dendritic cells, cancer cells, stem cells, and endothelial cells. They are now believed to be important for intercellular communication, but their functions remain elusive.

Exosomes are released either constitutively or in a regulated manner. For example, many tumor cells secrete exosomes in a constitutive manner [22], whereas B cells secrete detectable levels of exosomes upon activation of cell surface receptors [23]. Exosomes are formed by inward budding of late endosomes and produce multivesicular bodies that are then fused with the plasma membrane and released into the microenvironment [24]. Typically, exosomes have a diameter of 40–100 nm with a density of 1.13–1.19 g/mL in a sucrose solution, and can be sedimented by centrifugation at 100,000 g. In fact, exosomes are one type of microvesicles, a collective term for various types of membranous elements in the range of 20–1000 nm in diameter, which are released from and taken up by most cell types [25]. Other types or names are nanoparticles, microparticles, shedding microvesicles, apoptotic blebs, and human endogenous retroviral particles. Indeed, there are few firm criteria that distinguish one type of microvesicle from the other, and some researchers have termed exosomes as microvesicles in their articles.

### 3.2. Isolation, Storage Conditions, and Identification

#### 3.2.1. Isolation

The most common method used to isolate exosomes is ultracentrifugation that is often combined with sucrose density gradients or sucrose cushions. Cells and larger particles are removed by sequentially increasing the centrifugal forces, and then exosomes are precipitated by centrifugation at ≥100,000× *g* for at least 2 h [26]. This method provides highly enriched exosomes, but requires specialized equipment. Other methods include high-performance liquid chromatography (HPLC), ultrafiltration, and volume-excluding polymers. Isolation by HPLC allows exosomes to be preconcentrated by two filtration steps using 0.2-μm pore filters with a 100-kDa molecular weight cut-off after removal of the cells and larger particles by a low-gravity centrifugation step, followed by purification using size exclusion chromatography. To concentrate the eluted exosomes, the obtained fractions are typically centrifuged at ≥100,000× *g* during the final centrifugation step [27,28]. This method is complicated and seldom used to isolate exosomes. Isolation by ultrafiltration is based on the exosome size. Exosomes can be enriched with a commercially available nano-membrane concentrator by centrifugation at 3000× *g* for 10–30 min [29]. This method is less time consuming than ultracentrifugation and does not require the use of specialized equipment. In addition to these traditional methods, exosome isolation kits and exosome precipitation solutions have been developed by various companies in recent years. These products provide convenient and efficient techniques to isolate exosomes.

#### 3.2.2. Storage Conditions

Exosome sizes decrease by about 60% in storage at 37 ºC for 2 days. The size of exosomes does not change during the first two days of storage at 4 ºC. However, a decrease in size is observed after 3–4 days. When exosomes are stored at −20 ºC, their size remains constant over a long period. Notably, multiple cycles of deep-freezing and thawing do not affect the size of exosomes. Therefore, −20 ºC is a suitable temperature to store exosomes [27].

#### 3.2.3. Identification

Methods used to identify exosomes include scanning electron microscopy, atomic force microscopy, nanoparticle tracking analysis, transmission electron microscopy, flow cytometric analysis, western blotting, and enzyme-linked immunosorbent assay (ELISA) [27,30]. Two or three of these methods are usually used in combination for exosome identification. Exosomes are thought to be unique in their protein and lipid composition, providing some characteristics for their identification. First, they contain several adhesion molecules known to be expressed on the membranes of their parent cells; for example, CD80 and CD86 are expressed on dendritic cell-derived exosomes [31], and CD19 is expressed on B cell-derived exosomes [23]. In addition, because of their endosomal origin, all exosomes contain membrane transport and fusion proteins (GTPases, annexins, and flotillin), tetraspanins (CD9, CD63, CD81, and CD82), proteins involved in multivesicular body biogenesis (Alix and TSG101), as well as lipid-related proteins and phospholipases. These proteins can be detected by flow cytometric analysis, western blotting, and ELISA.

### 3.3. Components and Functions

The most unique function of exosomes might be specific interactions with targeted recipient cells, which putatively enables cell-to-cell communication between widely separated locations in the body [32]. Exosomes from various cell types have been implicated in important physiological and pathological processes such as disposal of unwanted proteins, antigen presentation, genetic exchange, immune responses, angiogenesis, inflammation, tumor metastasis, and spreading of pathogens or oncogenes [33,34], most of which are in accordance with the function of the cells of their origin. These functions are dependent on the components contained within the exosomes, especially proteins and RNAs.

#### 3.3.1. Proteins

The proteomes of exosomes have been analyzed by some research groups. About 1600 proteins have been found in biological fluid-derived exosomes, of which 300 are common in at least two sets and only two proteins are shared by more than four. Exosomes from cell culture media show higher homogeneity. Eight hundred and sixty proteins have been identified and about 110 of them are common in at least two sets and six proteins are shared by more than five [35]. Among these common proteins, in addition to cytoplasmic proteins, it is evident that there are a remarkable number of membrane proteins. Tetraspanins are most commonly associated with exosomes. These proteomic studies also indicate the possible physiological roles of exosomes. For example, exosomal proteins released by human cultured keratinocytes may function as extracellular matrix-modulating factors for dermal fibroblasts [36]. It has been estimated that, even though exosomes account for only a small proportion of the total plasma proteome, they are rich in proteins altered under a variety of pathological conditions. Therefore, they can also be regarded as diagnostic markers. In addition to these functions, a role of exosomes has been proposed in the migration of Dictyostelium cells through the secretion of chemoattractant signals [37].

#### 3.3.2. RNAs

In addition to protein delivery, exosomes transport mRNAs and miRNAs with the potential to alter the fate of recipient cells. Exosomes from glioblastoma cells are taken up by microvascular endothelial cells of the host human brain, resulting in translation of the mRNA molecules and stimulation of tubule formation by the recipient endothelial cells [38]. The levels of various mRNAs have been studied in exosomes derived from human breast milk. Certain miRNAs implicated in immune regulatory roles are present at high levels in the first 6 months of lactation, but are significantly reduced at later stages. It is thought that exosomes from breast milk modulate development of the infant immune system [39]. In addition, miRNAs secreted from Epstein Barr virus-infected cells are transferred by exosomes to uninfected recipient cells [40], indicating that exosomes have the potential to spread pathogens from one cell to another. Oncogenes can also be distributed by exosomes secreted from tumor cells [41]. This event leads to the transfer of oncogenic activity to target cells, which is an important factor in tumor metastasis.

Besides these functions, RNAs in exosomes may be useful diagnostic markers of various diseases. Specific expression patterns of serum miRNAs have been identified in lung cancer, colorectal cancer, and diabetes, providing evidence that serum miRNAs can serve as potential biomarkers for detection of various cancers and other diseases [42]. Many other body fluids have also been found to contain exosomes. Urine may be a very useful source of exosomal markers for urogenital diseases. Urinary exosomes contain mRNAs that are markers of renal ischemia/reperfusion injury [43] and prostate cancer [44]. Saliva-based diagnostics in addition to assessing the state of health of the oral cavity show the potential to monitor systemic health. Because of the reduced patient pain, convenience, greater speed, and lower cost of such analyses, several companies have initiated programs aimed toward exosome-based diagnostics, which show promising preliminary results.

#### 3.3.3. Other Components

In addition to proteins and RNAs, exosomes are enriched with certain raft-associated lipids such as cholesterol, ceramide, phospoglycerides, as well as long and saturated fatty-acyl chains. There are also indications that exosomes may deliver prostaglandins to target cells [45,46].

## 4. Properties of Exosomes Derived from MSCs

MSC-derived exosomes were first investigated in 2010 in a mouse model of myocardial ischemia/reperfusion injury [47] and were thereafter tested in several disease models. It has been tested that MSCs can produce higher amount of exosomes than other cells such as myoblast, the human acute monocytic leukemia cell line (THP-1) and the human embryonic kidney cell line (HEK), *etc.* [48]. There are no differences in terms of morphological features, isolation and storage conditions between exosomes derived from MSCs and other sources. As to the identification, MSC-derived exosomes express not only the common surface markers of exosomes, such as CD9 and CD81, but also some adhension molecules, including CD29, CD44 and CD73, which are expressed on the membrane of MSCs.

Similar to exosomes from other sources, protein components in MSC-derived exosomes do not remain constant when obtained from the conditioned media (CM) of various MSC batches. In three independent batches of MSC-derived exosomes, 379, 432, and 420 unique proteins have been detected by liquid chromatography-mass spectrometry/mass spectrometry, among which only 154 proteins are common [49]. Clustering of these proteins according to their functions suggested that exosomes have the potential to drive many biological processes. This notion is consistent with the reported efficacy of MSCs for the treatment of many diseases. Proteasome subunits have been reported to be present in MSC-derived exosomes [50]. Accumulation of misfolded proteins or oligomers is reduced in the heart tissues of a mouse model of myocardial ischemia/reperfusion injury, which has been treated with MSC-derived exosomes. Furthermore, the entire protein complement of a 20S proteasome has been detected with very high confidence by mass spectrometric analysis of MSC-derived exosomes. The 20S proteasome is responsible for degradation of intracellular oxidatively damaged proteins, which may partly contribute to the cardioprotective activity of MSC-derived exosomes [49].

MiRNAs contained in MSC-derived exosomes were also investigated. It has been found that miRNAs encapsulated in MSC-derived microparticles are predominantly in their precursor form [51]. Such microparticles show a hydrodynamic radius of 55–65 nm, which can be regarded as exosomes. MSCs may exert some biological effects on other cells through secretion of miRNAs in exosomes. Pretreating MSC-CM with RNase abolishes its renal protective effect completely [52]. Treatment of neurons and astrocytes with MSC-derived exosomes leads to an increase of miR-133b in these cells, which promotes functional recovery in Parkinson’s disease and spinal cord injury. This finding suggests that MSCs regulate neurite outgrowth at least partly by transferring miR-133b to neurons and astrocytes via the release of exosomes [53].

## 5. Applications of Exosomes Derived from MSCs

The information of recent research works on MSC-derived exosomes is summarized in Table 1.

### 5.1. Cardiovascular Disease

MSCs are the most widely applied stem cells in clinical trials of heart disease. An often-cited hypothesis is that transplanted MSCs differentiate into cardiomyocytes and supportive cell types to repair cardiac tissues. However, many observations are physically and temporally incompatible with the differentiation hypothesis [54], which has prompted an alternative hypothesis that transplanted MSCs mediate their therapeutic effect through secretion of paracrine factors that promote survival and tissue repair. Based on the paracrine hypothesis, researchers have begun to focus on the therapeutic effect of MSC-CM.

In a porcine model of myocardial ischemia and reperfusion (MI/R) injury, intravenous and intracoronary MSC-CM treatment reduces the infarct size by approximately 50% when administered just prior to reperfusion [55]. Size fractionation studies have demonstrated that the active component is a large complex of 50–200 nm in diameter. Electron microscopy showed that these complexes are phospholipid vesicles. They contain co-immunoprecipitating exosome-associated proteins CD81, CD9, and Alix, and can be purified with a hydrodynamic radius of 55–65 nm by size exclusion fractionation in HPLC. Purified exosomes administered to a mouse MI/R injury model revealed that MSCs mediate their cardioprotective paracrine effect by exosome secretion [47]. These results suggest that exosomes are a highly efficacious therapeutic agent that neutralizes MI/R injury and an effective adjuvant to complement current reperfusion therapy.

It is postulated that exosomes may participate in many biochemical and cellular activities and correct various ischemia-induced cascades. However, there are still some concerns regarding the use of exosomes for the treatment of MI/R injury. Many of the proteins in exosomes are enzymes, and the enzyme-based therapeutic activities may be activated by the release of injury-associated substrates. Resolution of the microenvironment would reduce the release of these substrates, which would influence enzymatic activities. Consequently, the efficacy of exosome-based therapeutics may be responsive to, but also limited by, the disease-precipitating microenvironment [56].

### 5.2. Kidney Injury

It has been suggested that MSCs may protect acute kidney injury experimental models from cisplatin, glycerol, and ischemia-reperfusion injury, but the mechanism is a matter of debate. Some studies have reported that transplanted MSCs infiltrate the kidney and directly repopulate the injured renal tubule [57]. Other studies have found no evidence of direct MSC engraftment in renal tubules during repair processes [58], suggesting paracrine effects of MSCs as the therapeutic modality [52]. It was later found that MSC-CM induces the migration and proliferation of kidney-derived epithelial cells, which diminishes tubule cell death *in vitro* and increases tubular cell survival, thereby limiting renal injury *in vivo*. These findings further indicate that tubular engraftment is unnecessary for the beneficial effects of MSCs [59].

To identify whether MSC-CM exerts a therapeutic effect through RNA or protein, CM pretreated with RNase or trypsin has been administered intravenously to rats with gentamicin-induced acute kidney injury. CM pretreated with RNase completely abolishes the protective effect, indicating the participation of RNA-like products in these processes [60]. It was further found that exosome-like microvesicles (average diameter: 100 nm) extracted from CM are the effective components. The protective effect of these microvesicles was also abolished by treatment with RNase, revealing that RNAs encapsulated in such microvesicles are the effective components in the treatment of kidney injury [52].

### 5.3. Immune Disease

Evidence from several studies has demonstrated that MSCs recruit and regulate T cells in both a cell-to-cell contact and a paracrine manner [61]. It is generally believed that secretion of inhibitory cytokines and inhibitory ligand-receptor interactions by MSCs play a key role in such functions. Based on this hypothesis, MSC-derived exosomes are viewed as potential mediators that induce peripheral tolerance toward autoreactive cells via bearing of tolerogenic molecules. Microvesicles (50–200 nm in diameter) isolated from MSC-CM have been co-cultured with splenic mononuclear cells isolated from a mouse model of experimental autoimmune encephalomyelitis. The results showed that MSC-derived microvesicles inhibit autoreactive lymphocyte proliferation and promote secretion of anti-inflammatory cytokines including interleukin (IL)-10 and transforming growth factor (TGF)-β. In addition, flow cytometric analysis showed that these MSC-derived microvesicles serve as vehicles for MSC-specific tolerogenic molecules such as PD-L1, Gal-1, and TGF-β. These observations suggest that MSC-derived exosomes are potent mediators that induce peripheral tolerance and modulate immune responses, which provide a new perspective towards indirect application of MSCs in the treatment of autoimmune diseases [30].

MSC-derived exosomes have also been tested in graft-versus-host disease (GVHD) recently. Subcutaneous injection of MSC-derived exosomes in mouse allogeneic skin grafting models delayed the occurence of GVHD for two days, which was concomitant with an increase in Tregs. *In vitro* studies revealed that exosomes activated MYD88-dependent signaling in monocytes to induce a M2-like phenotype, which polarized activated CD4^+^ T cells to Tregs [62].

However, incubation of exosomes from MSC-like cells derived from islets with non-obese diabetic (NOD) splenocytes results in the secretion of large amounts of IL-6, interferon-γ (IFN-γ), tumor necrosis factor-α, and monocyte chemoattractant protein-1, and activation of B cells, T cells, and antigen-presenting cells. At two weeks after intraperitoneal injection of exosomes into NOD mice, cells in the pancreatic and inguinal lymph nodes release large amounts of cytokines and chemokines in the absence of any additional antigens, together with a robust increase of IFN-γ-secreting cells and T-helper 1 cells. These results indicate that exosomes may be an autoantigen carrier with potent adjuvant activities and may function as an autoimmune trigger in NOD mice [63].

### 5.4. Tumor Growth

MSCs have contrasting effects on tumor growth because they are able to either favor tumor initiation or inhibit progression of established tumors. Factors produced by MSCs within the tumor microenvironment may be responsible for their various biological effects.

Both MSCs and MSC-derived exosomes increase the incidence and growth of SGC-7901 and SW480 cell-induced tumors, suggesting that MSC-derived exosomes promote tumor progression in a manner similar to that of MSCs *in vivo*. However, these MSC-derived exosomes have no significant effect on SGC-7901 cell proliferation *in vitro* [64]. Interestingly, treatment of SGC-7901 cells with a single dose of MSC-CM robustly induces tumor growth, whereas such a tumor-promoting activity cannot be effectively depleted by ultracentrifugation, suggesting that soluble proteins in MSC-CM are the active tumor-potentiating factors [65]. In another study, MSC-derived exosomes were found to suppress tumor progression and angiogenesis by down-regulating the expression of vascular endothelial growth factor (VEGF) in tumors *in vitro* and *in vivo*. MiR-16, a miRNA enriched in MSC-derived exosomes and known to target VEGF, was believed to be partially responsible for the anti-angiogenic effect [66].

### 5.5. Neurological Diseases

MSCs have a potential therapeutic benefit for the treatment of neurological diseases. They can interact with brain parenchymal cells to promote functional recovery. It has been hypothesized that MSCs communicate with parenchymal cells via miRNA contained in exosomes. In one study, when rats subjected to middle cerebral artery occlusion (MCAo) were treated with MSCs, microRNA 133b (miR-133b) levels in the ipsilateral hemisphere increased significantly. *In vitro*, miR-133b levels increased in exosomes derived from MSCs, which had been exposed to ipsilateral ischemic tissue extracts from rats subjected to MCAo. And miR-133b levels in primary cultured neurons and astrocytes treated with the exosome-enriched fractions released from these MSCs were also increased. However, this increase is significantly diminished by treatment of the astrocytes with exosome-enriched fractions from MSCs transfected with an miR-133b inhibitor. This experiment provides the first evidence that MSCs communicate with brain parenchymal cells via exosome-mediated miR-133b transfer, leading to regulation of specific gene expression that enhances neurite outgrowth and functional recovery [53]. In another study of the same research group, the authors demonstrated that intravenous injection of MSC-derived exosomes can lead to the increase of axonal density and synaptophysin-positive areas along the ischemic boundary zone of the cortex and striatum and prompt functional recovery in the same model as above, confirming that exosomes from MSCs could significantly improve neurologic outcome and contribute to neurovascular remodeling [67].

The accumulation of β-amyloid (Aβ) peptides in the brain is a characteristic of Alzheimer’s disease. Furthermore, neprilysin (NEP) is the most important Aβ-degrading enzyme in the brain. It has been found that adipose tissue-derived mesenchymal stem cells (ADSCs) secrete functional NEP in association with exosomes. Co-culture of N2a neuroblastoma cells overexpressing human Aβ and ADSCs results in a significant decrease of both Aβ40 and 42 levels in the culture medium. Moreover, it has been demonstrated that ADSCs express higher levels of NEP than those in bone marrow-derived MSCs. These results suggest that ADSCs may serve as a promising cell source for exosome-based Alzheimer’s disease treatments [68].

## 6. Conclusions

There have been some encouraging therapeutic effects of MSC-derived exosomes in various animal models. It has been suggested that exosomes are ideal vehicles for drug delivery, because they encapsulate prolific proteins and RNAs, can cross the plasma membrane to deliver their cargo into target cells and are well tolerated by the body [69]. MSCs are prolific producers of exosomes. They can produce high amounts of exosomes, and the production is not compromised in terms of quantity and quality by immortalization of cells to generate permanent cell lines, thus ensuring sustainable and reproducible production of exosomes from MSCs [48]. In addition, accumulating evidence has suggested that the exosome secretion profile can be improved by preconditioning or genetic manipulation of the parent cells [70,71]. Considering that genetically modified MSCs were widely tested in various studies [72,73], we hypothesize that exosomes derived from pretreated MSCs could be used as ideal vehicles for drug or gene delivery to facilitate gene and cell therapy. Therefore, given the merits of MSCs, exosomes hold great promise as a controllable, manageable, and feasible approach in future studies.

However, based on the proteomic and genomic complexities of exosomes, their possible mechanisms and exact compositions need further investigation. Because the protein and RNA components are not always the same in exosomes obtained from CM of MSCs cultured for different growth periods, the collection protocol should be standardized to cater to the needs of different studies.

## Figures and Tables

**Table 1.** Information of MSC-derived exosomes in different studies.

Article	Name and size (nm)	Isolation method	Identify method	Origin	Delivery way	Biological function
Mokarizadeh (2012) [30]	Microvesicle 50–200	ultracentrifugation (100,000 *g* 2 h)	Flowcytometry and electron microscopy	murine BMSC	*in vitro* coculture	Induce peripheral tolerance
Lai (2010) [47]	Exosome 55–65	ultracentrifugation (100,000× *g* 1 h) HPLC	Flowcytometry	HESC-derived MSC	intravenous injection	Reduces myocardial ischemia/reperfusion injury
Reis (2012) [52]	Exosome-like microvesicle <100	ultracentrifugation (100,000× *g* 1 h)	Electron microscopy	rat BMSC	intravenous injection	Repaired gentamicin induced acute kidney injury
Zhang (2013) [62]	exosome	ultrafiltration HPLC	Not shown	HESC-derived MSC	subcutaneous injection	Enhance survival of allogeneic skin graft and increase Tregs
Rahman (2013) [63]	Exosome not shown	ultracentrifugation (100,000× *g* 1 h)	Flowcytometry and electron microscopy	islet MSC-like cells	intraperitoneal injection	Trigger autoimmune response in NOD mice
Zhu (2012) [64]	Exosome 30–100	ultrafiltration ultracentrifugation (100,000× *g* 1 h)	Western blotting	hBMSC	subcutaneous injection	Promote tumor growth *in vivo*
Lee (2013) [66]	Exosome not shown	ExoQuick-TC (System Bioscience, Mountain View, CA, USA)	Western blotting	murine BMSC	subcutaneous injection	Inhibit angiogenesis
Xin (2013) [67]	Exosome not shown	ultracentrifugation	Not shown	rat BMSC	intravenous injection	Promote neurovascular remodeling and functional recovery after stroke
